# Choice of Mouse Strain Influences the Outcome in a Mouse Model of Chemical-Induced Asthma

**DOI:** 10.1371/journal.pone.0012581

**Published:** 2010-09-07

**Authors:** Vanessa De Vooght, Jeroen A. J. Vanoirbeek, Katrien Luyts, Steven Haenen, Benoit Nemery, Peter H. M. Hoet

**Affiliations:** Research Unit Lung Toxicology, Katholieke Universiteit Leuven, Leuven, Belgium; Ludwig-Maximilians-Universität München, Germany

## Abstract

**Background:**

The development of occupational asthma is the result of interactions between environmental factors and individual susceptibility. We assessed how our model of chemical-induced asthma is influenced by using different mouse strains.

**Methodology/Principal Findings:**

On days 1 and 8, male mice of 7 different strains (BALB/c, BP/2, A/J, C57Bl/6, DBA/2, CBA and AKR) were dermally treated with toluene-2,4-diisocyanate (TDI) (0.3%) or vehicle (acetone/olive oil, AOO, 2∶3) on each ear (20 µl). On day 15, they received an oropharyngeal instillation of TDI (0.01%) or AOO (1∶4). Airway reactivity to methacholine, total and differential cell counts in bronchoalveolar lavage (BAL) and total serum IgE and IgG_2a_ levels were measured. Lymphocyte subpopulations in auricular lymph nodes and *in vitro* release of cytokines by ConA stimulated lymphocytes were assessed. In TDI-sensitized and challenged mice, airway hyper-reactivity was only observed in BALB/c, BP/2, A/J and AKR mice; airway inflammation was most pronounced in BALB/c mice; numbers of T-helper (CD4^+^), T-activated (CD4^+^CD25^+^), T-cytotoxic (CD8^+^) and B- lymphocytes (CD19^+^) were increased in the auricular lymph nodes of BALB/c, BP/2, A/J and CBA mice; elevated concentrations of IL-4, IL-10, IL-13 and IFN-γ were detected in supernatant of lymphocytes from BALB/c, BP/2, A/J, C57Bl/6 and CBA mice cultured with concanavaline A, along with an increase in total serum IgE.

**Conclusion:**

The used mouse strain has considerable and variable impacts on different aspects of the asthma phenotype. The human phenotypical characteristics of chemically-induced occupational asthma were best reproduced in Th2-biased mice and in particular in BALB/c mice.

## Introduction

Asthma is a chronic inflammatory airway disease that has emerged as a major health concern among industrialized nations [1]. In adult asthma, occupational exposures are estimated to be responsible for 10% to 25% of the asthma prevalence [2]. Occupational asthma has become the most common work-related lung disease.

Diisocyanates, used in the production of polyurethanes and one of the leading causes of occupational asthma worldwide, are highly reactive low molecular weight chemicals with significant sensitizing potential [3], [4].

Although exposed to similar allergic stimuli, not all humans develop allergic or occupational asthma. The etiology of asthma is complex and is associated with a combination of immune, genetic, environmental and socio-economic factors [1], [5].

The mouse genome has been extensively characterized and exhibits a high degree of homology with its human counterpart. Especially, a high level of resemblance at the immunological level has been found, suggesting that mouse models can yield a relevant insight into human pathology [6]. Maximum genetic uniformity can be achieved when using inbred strains. Environmental factors such as exposure to light, diet, air quality and climate can influence the phenotypic outcome of asthma. The advantage of using an animal model is that these confounding factors can be largely controlled in laboratory animal housing situations [5], [7]. Using different inbred strains of mice gives the opportunity to estimate the influence of the genetic background on the phenotypical outcome of chemical-induced asthma.

Starting from an established mouse model of chemical-induced asthma developed by Vanoirbeek *et al.* in BALB/c mice, 6 other inbred mouse strains were tested on their sensitizing capacity to toluene-2,4-diisocyanate (TDI) [8]–[12]. Three of the selected mouse strains are Th2-biased (BALB/c, BP2 and A/J), while the others were Th1-biased strains (C57Bl/6, DBA/2, AKR and CBA). By testing different mouse strains we have the opportunity to evaluate the intra- (experimental) and the inter-strain variability, giving us the possibility to select the inbred mouse strain which represents best the phenotype of human occupational asthma.

## Materials and Methods

### Ethics statement

All experimental procedures were approved by the local Ethical Committee for Animal Experimentation of the K.U.Leuven (project license number 072/2009). The research projects that are approved by the local Ethical Committee for Animal Experimentation of the K.U.Leuven are in accordance with the Belgian and European laws, guidelines and policies for animal experimentation, housing and care. This means that they are in accordance with the Belgian Royal Decree of 14 November 1993 concerning the protection of laboratory animals and the European Directive 86-609-EEC. The animals are housed – according to the Belgian and European laws, guidelines and policies for animal experiments, housing and care - in the Central Animal Facilities of the university. These facilities have the obligatory accreditation of the authorized Belgian Ministry and are registered under license number LA2210393. All efforts were made to minimize suffering.

### Reagents

Toluene-2,4-diisocyanate (98%, CAS 584-84-9) (MW: 174.15), acetyl-β-methylcholine (methacholine), acetone and diethyl ether were obtained from Sigma-Aldrich (Bornem, Belgium). Isoflurane (Forene®) was obtained from Abbott Laboratories (S.A. Abbott N.V., Ottignies, Belgium) and Pentobarbital (Nembutal®) from Sanofi Santé Animale (CEVA, Brussels, Belgium). For dermal applications, the vehicle (AOO) consisted of 2 volumes of acetone (A) and 3 volumes of olive oil (OO) (Selection de Almazara, Carbonell, Madrid, Spain). For the oropharygeal aspiration the vehicle (AOO) consisted of a mixture of 1 volume of acetone and 4 volumes of olive oil. AOO is one of the standard vehicles for the local lymph node assay (OECD 2002, guideline 429) and it has been proven to be an ideal vehicle for non-aqueous compounds like TDI. In previous experiments we optimized the proportions of A and OO for dermal sensitization and challenge to avoid irritation, inflammation or airway response, in the control group [8], [12]. Concentrations of TDI are given as percent (v/v) in AOO.

### Animals

Male BALB/c OlaHsd, A/J OlaHsd, C57Bl/6J OlaHsd, AKR OlaHsd, DBA/2 OlaHsd and CBA/J CrHsd mice (6 weeks old) were obtained from Harlan (Horst, The Netherlands), while male BP/2IcRj-Biozzi mice were purchased from Janvier (Le Genest Saint Isle, France). The mice were housed in a conventional animal house with 12-h dark/light cycles. They were housed in filter top cages and received lightly acidified water and pelleted food (Trouw Nutrition, Gent, Belgium) *ad libitum*.

### Groups of animals and treatment protocol

As previously described, on days 1 and 8, the animals received a dermal application (20 µl) of 0.3% TDI or vehicle (AOO, 2∶3) on the dorsum of both ears. On day 15, they received, under light isoflurane anesthesia, an oropharyngeal aspiration (20 µl) of 0.01% TDI (challenge) or vehicle (AOO, 1∶4) [12]. Mice were sacrificed 24 hours after the last challenge. Each group consisted of 6–14 animals. Experimental groups are 0/0, 0/1 and 1/1. The first digit identifies the agent used for the dermal application on days 1 and 8 (sensitization) and the second digit identifies the agent administered via oropharyngeal aspiration on day 15 (challenge). Treatment with TDI is indicated as 1 and treatment with vehicle is indicated as 0. Thus, the 1/1 group, for example, consists of mice that received dermal applications of TDI (days 1 and 8, first digit) and an oropharyngeal challenge of TDI (day 15, second digit). All treatments and procedures were performed each time around the same hour of the day and the different experimental groups (0/0, 0/1 and 1/1) were mixed within each mouse strain. Different strains were performed in succession.

### Airway hyper-reactivity (AHR)

Airway hyper-reactivity was measured 24 h after the oropharyngeal challenge, using a forced oscillation technique (FlexiVent, SCIREQ, Montreal, Canada). As previously described, mice were anesthetized with pentobarbital (70 mg/kg body weight) and airway resistance (R) to increasing concentrations of methacholine (from 0 to 10 mg/ml) was measured using a “snapshot” protocol. For each mouse, R was plotted against methacholine concentration (from 0 to 10 mg/ml) and the area under the R-methacholine concentration curve (AUC) was calculated [13].

### Pulmonary inflammation (bronchoalveolar lavage)

After the functional airway measurements (1 day after challenge), mice were deeply anesthetized by an intraperitoneal injection of pentobarbital (90 mg/kg body weight). For later measurements of total serum immunoglobulins, blood was first sampled from the retro-orbital plexus, and the mice were then killed by section of the abdominal vessels. Serum samples, obtained after centrifugation (14,000 g, 10 min) of whole blood, were stored at −80°C until analysis. The lungs were lavaged, *in situ*, three times with 1 ml sterile saline (0.9% NaCl) and kept on ice. The recovered fluid of the first lavage was kept separately, while the two subsequent lavages were pooled. The first lavage was centrifuged (1000 g, 4°C, 10 min) and the supernatant was frozen (−80°C) for further analyses of cytokines, while the pellet was resuspended with 0.9% NaCl to match the original volume. Subsequently, all three lavages were pooled and the cells were counted using a Bürker hemocytometer (total cell count) to assess airway inflammation. For differential cell counts, cells were spun (300 g, 6 min) (Cytospin 3, Shandon, TechGen, Zellik, Belgium) onto microscope slides, air-dried and stained (Diff-Quik® method, Medical Diagnostics, Düdingen, Germany). For each sample, 200 cells were counted for the number of macrophages, eosinophils, neutrophils and lymphocytes.

Levels of IL-2, IL-5, IL-6, IL-13, tumor necrosis factor-alpha (TNF-α), and cytokine-induced neutrophils chemoattractant (KC) were measured in undiluted BAL fluid (first fraction) by Cytometric Bead Array and were analyzed with FCAP Array Software on the FACSArray (BD Bioscience, Erembodegem, Belgium). Lower limits of detection were 0.2 pg/ml, 0.9 pg/ml, 1.4 pg/ml, 1.4 pg/ml, 0.8 pg/ml and 2.4 pg/ml, respectively.

### Lymph node cells analysis

To characterize the different subpopulations of lymphocytes and to measure cytokines in the supernatant of cultured lymphocytes, retro-auricular lymph nodes were obtained from the same mice and were processed for each mouse separately, except when there were not enough cells present to perform FACS analyses and cell culture in which case lymph nodes of different mice were pooled. The lymph nodes were kept on ice in RPMI-1640 (Invitrogen, Merelbeke, Belgium) and cell suspensions were obtained by pressing the lymph nodes through a cell strainer (100 µm) (BD Bioscience, Erembodegem, Belgium) and rinsing with 10 ml tissue culture medium (RPMI-1640). Cells were counted using a Bürker hemocytometer. Lymphocytes were washed three times and suspended (10^7^ cells/ml) in complete tissue culture medium (RPMI-1640 supplemented with 10% heat-inactivated fetal bovine serum, 10 mg/ml streptomycin and 100 IU/ml penicillin). Five-hundred thousand cells were stained with anti-CD3^+^ (APC, T-lymphocytes), anti-CD4^+^ (APC-Cy7, Th-lymphocytes), anti-CD8^+^ (PerCP-Cy5.5, Tc-lymphocytes) and anti-CD25^+^ (PE, activated/regulatory T-lymphocytes), or received a single staining with anti-CD19^+^ (PE, B-lymphocytes) labeled antibodies, according to standard procedures, and with control samples being labeled with isotype match control antibodies (BD Biosciences, Erembodegem, Belgium). Flow cytometry (FACSArray, BD Biosciences, Erembodegem, Belgium) was performed using at least 10^5^ cells.

Cells were seeded into 48-well culture plates at a density of 10^6^ cells/ml and incubated in complete RPMI-1640 medium for 42 h with 2.5 µg/ml of concanavaline A (ConA) (Sigma–Aldrich, Bornem, Belgium). Cells were centrifuged (1000 g, 4°C, 10 min) and supernatants were stored at −80°C. Levels of IL-2, IL-4, IL-10, IL-13, IL-17 and interferon gamma (IFN-γ) were measured via Cytometric Bead Array and analyzed with the FCAP Array Software on the FACSArray (BD Biosciences, Erembodegem, Belgium). Lower detection limits were 0.2 pg/ml, 0.3 pg/ml, 9.6 pg/ml, 2.4 pg/ml and 0.95 pg/ml and 0.5 pg/ml, respectively.

### Total serum IgE and IgG_2a_

The OptEIA™ Mouse IgE and IgG_2a_ set from Pharmingen (BD Biosciences, Erembodegem, Belgium) were used to measure total serum IgE (diluted 1/70) and total serum IgG_2a_ (diluted 1/10,000). Measurements were performed according to the manufacturer's instructions.

### Data analysis

A normal distribution of the data was assessed by the D'Agostino & Pearson omnibus normality test. All data are presented as means and standard deviations (S.D.), except for the AUC data of the AHR, which is shown as individual data and group means. The concentrations of cytokines in BAL and supernatant, as well as the lymph node subpopulations and IgE and IgG_2a_ were analyzed with a one-way ANOVA test followed by a Bonferroni's multiple comparison test. According to the D'Agostino & Pearson omnibus normality test, the AUC data of the AHR and the differential cell counts were not normally distributed and therefore were analyzed with a non-parametric Kruskal–Wallis test followed by a Dunn's multiple comparison test (Graphpad Prism 4.01, Graphpad Software Inc, San Diego, USA). A level of p<0.05 (two-tailed) was considered significant.

## Results

**Figure 1:**
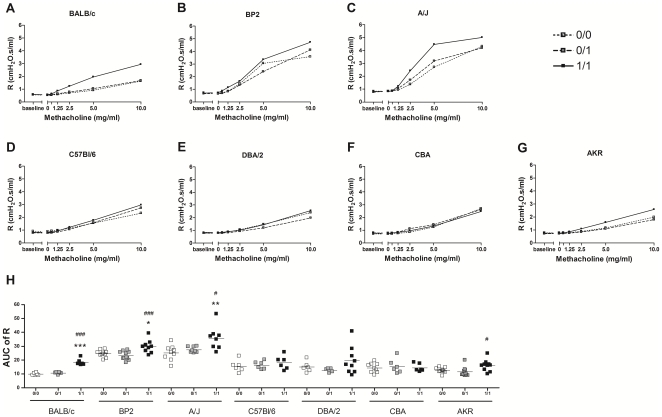
Airway hyperresponsiveness (AHR) in different mouse strains. The airway resistance (R), after increasing concentrations of methacholine (0–10 mg/ml), was measured 24 hours after the challenge. Figures A) to G) reflect the airway hyperresponsiveness to increasing concentrations of methacholine per mouse strain. Figure H) represents the AUC of R per experimental condition and per mouse strain. Experimental groups are 0/0, 0/1 and 1/1. The first number identifies the agent used for the dermal applications (sensitizations) and the second number identifies the agent used for the oropharyngeal aspiration (challenge). A treatment with toluene-2,4-diisocyanate (TDI) is shown as 1 and a treatment with the vehicle (a mixture of acetone and olive oil), is shown as 0. Data are presented as mean ± S.D. (A–G) and mean with individual values (H), n = 5–11 per group, * p<0.05, ** p<0.01 and *** p<0.001 compared with the 0/0 group, # p<0.05 and ### p<0.001 compared with the 0/1 group.

Figure 1 shows the results of airway reactivity to methacholine 24 hours after the challenge with TDI or vehicle. Figures 1A to 1G show the airway resistance (R) after increasing concentrations of methacholine for the different mouse strains separately, while figure 1H shows the AUC of the R against methacholine concentration. Significant differences between the TDI-treated and vehicle-treated animals were found in all the Th2-biased strains (BALB/c, BP2 and A/J) and also in one of the Th1-biased strains (AKR). BALB/c mice exhibited the most pronounced difference between the 1/1 group and the two control groups.

**Figure 2:**
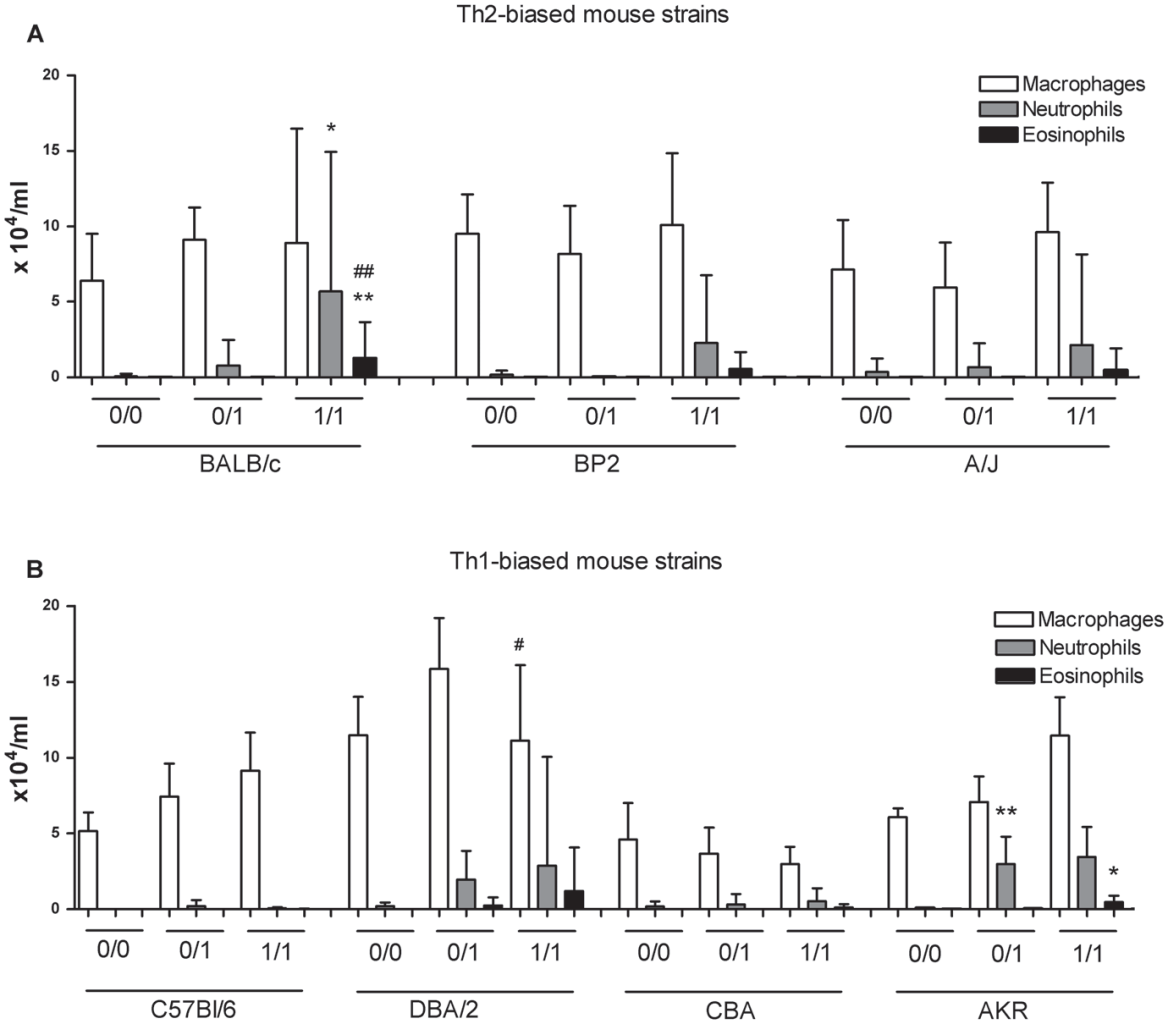
Differential cell count in BAL in different mouse strains. The number of macrophages, neutrophils and eosinophils was counted in the BAL fluid. Figure A) represents the differential cell count in the Th2-biased mouse strains and figure B) the differential cell count in the Th1-biased mouse strains. Experimental groups are identical to figure 1. Data are presented as means ± S.D., n = 6−14 per group, * p<0.05 and ** p<0.01 compared to the 0/0 group, # p<0.05 and # p<0.01 compared to the 0/1 group.

The numbers of macrophages, neutrophils and eosinophils in BAL fluid, 24 hours after challenge, are is shown in figures 2A and 2B. BALB/c mice exhibited significant increases in neutrophils (6-fold) and eosinophils (1.3-fold) after sensitization and challenge with TDI compared to the 0/0 group. Among the Th-1 biased animals, the AKR mice also exhibited significant increases in neutrophil and eosinophil numbers.

The cytokines IL-2, IL-5, IL-6, IL-13, TNF-α and KC were assessed in the BAL fluid (data not shown). In none of the mouse strains were there any significant differences between the 1/1 groups and their respective control groups (0/0, 0/1).

**Figure 3:**
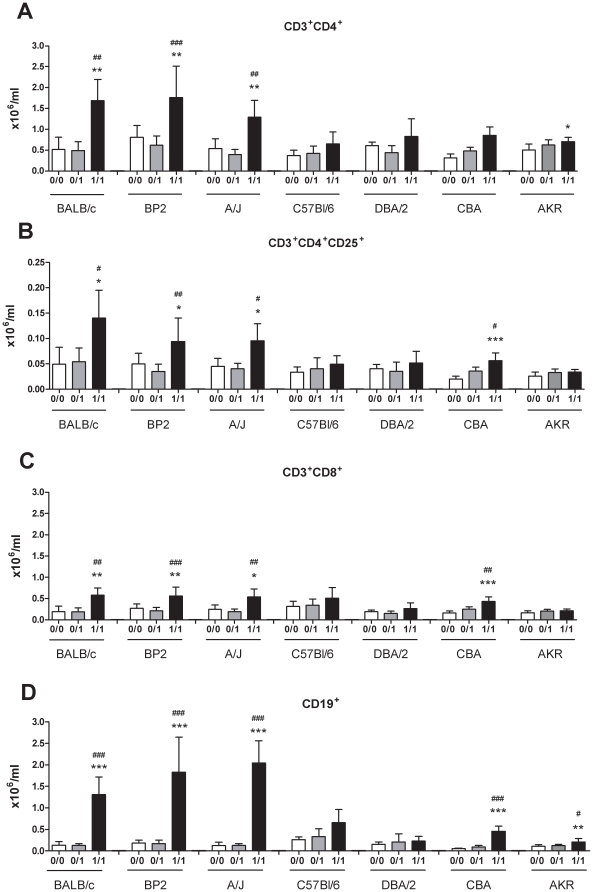
Lymphocyte subpopulations in the auricular lymph nodes of different mouse strains. Auricular lymph nodes were collected and FACS analyses were performed. A) CD3^+^CD4^+^ (Th-lymphocytes), B) CD3^+^CD4^+^CD25^+^ (activated/Treg-lymphocytes), C) CD3^+^CD8^+^ (Tc-lymphocytes) and D) CD19^+^ (B-lymphocytes) lymphocytes were characterized. Experimental groups are the same as in figure 1. Data are presented as means ± S.D., n = 4−9, * p<0.05, ** p<0.01 and *** p<0.001 compared to the 0/0 group, # p<0.05, ## p<0.01 and ### p<0.001 compared to the 0/1 group.

Lymphocyte subpopulations were characterized in auricular lymph nodes, 24 hours after the challenge (figure 3). All the Th2-biased strains (BALB/c, BP2 and A/J) exhibited a significantly higher amount of Th-lymphocytes (CD3^+^CD4^+^, figure 3A), activated/Treg-lymphocytes (CD3^+^CD4^+^CD25^+^, figure 3B), Tc-lymphocytes (CD3^+^CD8^+^, figure 3C) and B-lymphocytes (CD19^+^, figure 3D) in TDI sensitized and challenged animals compared to their control groups. Among the Th1-biased strains, significant differences in Treg-, Tc- and B-lymphocytes were found in CBA mice, and AKR mice showed significantly higher amounts of Th- and B-lymphocytes in the 1/1 group compared to the controls.

Table 1 gives an overview of the different cytokines measured in the supernatants of auricular lymphocytes cultured in the presence of ConA. IL-2 and IL-17 gave no significant differences in any of the mouse strains except for an increase in IL-2 in the 1/1 group of the C57Bl/6 compared to the 0/0 group (data not shown). IL-4 levels are significantly higher in the 1/1 groups of all mouse strains, except in C57Bl/6 mice. Increased concentrations of IL-10 were found for BALB/c, BP2, A/J, DBA/2 and AKR mice, whereas IL-13 was increased in all Th2-biased mice and in AKR mice. Concentrations of IFN-γ were significantly increased in BALB/c, BP2 and A/J mice.

**Table 1:** Cytokines in supernatants of lymphocytes obtained from auricular lymph nodes.

		IL-4	IL-10	IL-13	IFN-γ
**BALB/c**	**0/0**	1.3±0.3	29.9±12,0	8.1±4,0	103±41
	**0/1**	0.9±0.1	22.4±20,0	8.7±11,0	124.1±68,0
	**1/1**	10.1±7,0^(*, ##)^	110±42^(**, ##)^	162±58^(***, ###)^	457±188^(**, ##)^
**BP2**	**0/0**	0.2±0.6	5.6±8,0	10.8±14,0	441.1±668,0
	**0/1**	0.2±0.2	3.7±3,0	7.3±4,0	390±314
	**1/1**	4±2^(***, ###)^	46.6±32^(**,##)^	530±249^(***,###)^	2251±1316^(**, ##)^
**A/J**	**0/0**	0.3±0.4	3.1±4,0	4.1±4,0	123.5±15,0
	**0/1**	0.4±0.2	4.2±2,0	3±2	86.7±59,0
	**1/1**	8.2±3^(***, ###)^	77.3±69,0^(*)^	226±159^(*, #)^	809±542*
**C57Bl/6**	**0/0**	1.4±1,0	49.5±61,0	90.3±120,0	78.4±94,0
	**0/1**	0.6±0.5	16.3±14,0	45.4±47,0	170.5±290,0
	**1/1**	1.4±1,0	46.2±40,0	106.7±86,0	296.5±511,0
**DBA/2**	**0/0**	0.8±0.3	13.9±7,0	14.7±10,0	433.2±174,0
	**0/1**	1.3±0.5	28.4±17,0	28.6±20,0	470.6±226,0
	**1/1**	5.2±2,0^(**, ##)^	46.7±19,0^(**)^	59.4±39,0	755±428
**CBA**	**0/0**	1.3±0.5	15.5±8,0	16±9	985.8±438,0
	**0/1**	0.5±0.1	3.1±2,0	2±2	142.2±110,0
	**1/1**	7.9±2^(***, ###)^	63.5±20,0^(##)^	184.1±62,0^(###)^	836.7±231,0
**AKR**	**0/0**	0.6±0.2	54.8±28,0	56.7±36,0	3124±683
	**0/1**	0.6±0.2	46.7±29,0	54.6±22,0	3286±373
	**1/1**	2.4±2,0^(*, #)^	115.3±51^(*, #)^	231±93^(**, ##)^	3383±104

Auricular lymph node cells were cultured (42 h) with concanavaline A (2.5 µg/ml). Concentrations (pg/ml) of IL-2 (data not shown), IL-4, IL-10, IL-13, IL-17 (data not shown) and IFN-γ were measured, by Cytometric Bead Array, in the supernatant. Experimental groups are identical to those in figure 1. Data are presented as mean ± S.D., n = 4−11 values per group. * p<0.05, ** p<0.01 and *** p<0.001 compared to the 0/0 group and ^#^ p<0.05, ^##^ p<0.01 and ^###^ p<0.001 compared to the 0/1 group.

**Figure 4:**
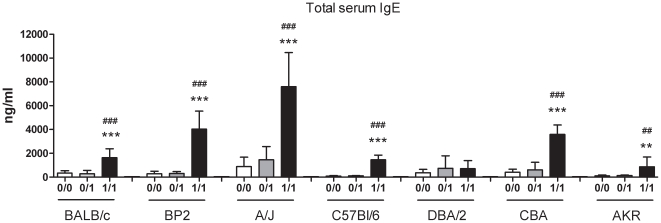
Total serum IgE in different mouse strains. Total serum IgE was measured 24 hours after the challenge. Experimental groups are the same as in figure 1. Data are presented as means ± SD, n = 6–14, ** p<0.01 and *** p<0.001 compared to the 0/0 group, ## p<0.01 and ### p<0.001 compared to the 0/1 group.

Total serum IgE levels (figure 4) were significantly higher compared to their two control groups (0/0, 0/1) in all mouse strains, except DBA/2 mice. No differences were found for total serum IgG_2a_ levels (data not shown).

## Discussion

Seven mouse strains were tested in an established mouse model of chemical-induced asthma [8]–[12]. The main findings of this study were that, in general, Th2-biased mice reproduce better than Th1-biased mice, the features that characterize human occupational asthma. BALB/c mice showed the most pronounced differences in AHR, airway inflammation and immunologic parameters, when compared to the two other Th2-biased mouse strains tested (BP2 and A/J). Th1-biased mice, however, were not completely non-responsive.

Mice are the most commonly used species to develop experimental models of human diseases. Mice are easy to breed, economical to house and relatively easy to work with. Furthermore, the mouse genome has been extensively studied and it exhibits a high degree of homology with the human genome. Further advantages are the wide variety of available immunological and molecular reagents as well as transgenic animals [6], [14].

Besides the many advantages of animal models, there is still a lot of controversy concerning the use of animals to study human disease. Thus, mice do not spontaneously exhibit symptoms consistent with asthma. Different treatment protocols have been developed to mimic the phenotypes of human asthma, but no mouse model is currently able to mimic the full range of clinical manifestations. Furthermore, important differences exist in airway development and morphology between humans and mice. Mouse airways have fewer airway generations and do not contain smooth muscle bundles. Nevertheless, although mice cannot be considered as perfect surrogates for humans, they can be used to test hypotheses in a relatively simple controlled system [6], [15], [16].

In view of the above considerations, it is important to standardize techniques and protocols used, to be able to compare the results of different research groups. So far, the influence of the genetic background of mice on ventilatory and immunological parameters has not been studied much. Our study is one of the first to have compared multiple endpoints of immune-mediated asthma in a large number of inbred mouse strains. It is surprising how little is known about genetic differences between the commercially available inbred mouse strains, which makes it hard to link phenotypic differences in parameters to genetic variability. So far, our ventilatory, inflammatory and immunologic results can only be linked to a predominant Th1- or Th2-bias.

Clear differences were found in airway hyper-reactivity to methacholine in the different mouse strains. Th2-biased mouse strains showed increases in airway hyper-reactivity compared to Th1-biased mice, as already shown by different other research groups in other asthma models [1], [17]–[20]. However, one exception is the AKR strain, which although being Th1-biased, responded significantly in terms of increase in airway hyper-reactivity [21]. Furthermore, we found differences in baseline reactivity to methacholine between the different Th2-biased mice. BP2 and A/J mice were found to be more sensitive to methacholine provocation than BALB/c mice and this both for the TDI-treated animals and the control mice. Among the three Th2-biased strains, the BALB/c mice presented the best separation between TDI-sensitized plus TDI-challenged animals and the controls. Differences in baseline airway hyper-reactivity can be an intrinsic characteristic of the mouse strain. Differences in alveolar size, lung volume, elastic properties and differences in controlling smooth muscle cells by the autonomic nerve system have been described [22]–[24].

When we analyse our results of the airway hyper-reactivity more in detail, we can conclude that individual adjustments for methacholine concentrations per mouse strain are preferable. A/J mice reach a plateau at 10 mg/ml methacholine (figure 1C), while C57Bl/6 mice are still at a submaximum at that concentration (figure 1D). We chose to perform the methacholine provocation with the same methacholine concentrations for each mouse strain to make comparisons easier.

In our model, airway inflammation in TDI-induced asthma is characterized by an influx of mainly neutrophils and also some eosinophils [10], [12], [25]. This study showed that BALB/c mice have the most pronounced airway inflammation of all 7 mouse strains. However, our observations support that there is no consistent relationship between airway hyper-reactivity and the influx of neutrophils and eosinophils in the lungs, as already shown by Whitehead *et al.* [26]. When using ovalbumin models, C57Bl/6 are often used and they respond with a robust airway eosinophilic response [19], [27]. It is conceivable, as suggested by Herrick *et al.*, that the ability to generate airway inflammation after chemical exposure is under different and perhaps tighter genetic control than the ability to mount these responses after exposure to other antigens [27].

Significant increases in the total amount of Th-, Treg-, Tc- and B-lymphocytes were found in all Th2-biased mouse strains and for some lymphocyte subpopulations also for the Th1-biased CBA and AKR mice. Vogelsang *et al.* already showed that there are different amounts of conventional and plasmacytoid dendritic cells and Treg-lymphocytes in blood and spleen of BALB/c vs. C57Bl/10J mice [28]. This confirms that the genetic background of the mouse strain plays a role in the development of different immunologically linked cell subtypes and probably also in their cytokine production.

Increases in IL-4 were associated with increases in the total amount of B-lymphocytes and with proportional increases in total serum IgE. However, BALB/c mice turned out to be limited producers of total serum IgE compared to the other mouse strains tested. This raises again the question of the relevance of IgE in chemical-induced asthma. For many low molecular weight compounds, specific IgE antibodies have only been found in a small subset of patients [29]. The presence of total IgE in serum serves as a marker of prior sensitization in mice, but has limited functional consequences. This confirms the hypothesis of a non-IgE mediated cellular mechanism involved in the development of chemical-induced asthma. In our data the amount of IL-4 does not always correlate with the level of IgE present in the serum. IL-4 plays an important role in the production of IgE, but mediates also other pathways such as the differentiation of Th-lymphocytes, the ability to induce the expression of vascular cell adhesion molecule 1 (VCAM-1) and the production of mucus in the asthmatic airway [30].

**Figure 5:**
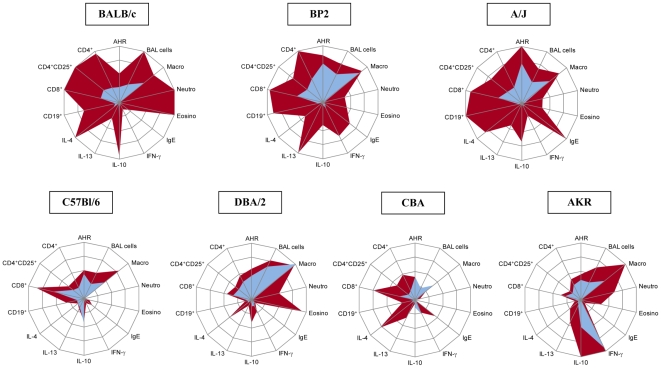
Radar graphs of the different mouse strains. The radar graphs give a visual overview of all results combined for the complete control group (0/0) (blue field) versus the complete TDI-treated group (1/1) (red field). Experimental groups are the same as in figure 1. The lower limit of each axis is always 0. The upper limit of each axis is the maximum average for a specific parameter measured in one strain and is presented as 100%. AHR  =  area under the curve (AUC) of the airway hyper-reactivity (0–36 AUC of airway resistance) (figure 1H); BAL cells  =  total BAL cell count (0–18.2×10^4^ cells); Macro  =  total number of BAL macrophages (0–11.5×10^4^ cells) (figure 2A and B); Neutro  =  total number of BAL neutrophils (0–5.7×10^4^ cells) (figure 2A and B); Eosino  =  total number of BAL eosinophils (0–1.3×10^4^ cells) (figure 2A and B); IgE  =  total serum IgE (0–8000 ng/ml) (figure 4); IFN-γ, IL-10, IL-13 and IL-4  =  cytokines measured in supernatant of cultured auricular lymphocytes (see table 1), IFN-γ (0–3400 pg/ml), IL-10 (0–116 pg/ml), IL-13 (0–530 pg/ml) and IL-4 (0–10.2 pg/ml); CD19^+^  =  CD19^+^ B-lymphocytes per auricular lymph node (0–2.1×10^6^ cells) (figure 3D); CD8^+^  =  CD3^+^CD8^+^ Tc-lymphocytes per auricular lymph node (0–0.6×10^6^ cells) (figure 3C); CD4^+^CD25^+^  =  CD3^+^CD4^+^CD25^+^ activated/Treg-lymphocytes per auricular lymph node (0–0.145×10^6^ cells) (figure 3B); CD4^+^  =  CD3^+^CD4^+^ Th-lymphocytes per auricular lymph node (0–1.8×10^6^ cells) (figure 3A). For specific significant differences between the 0/0 and the 1/1 group, check the specific graphs.

In an attempt to create a comprehensible overview of all the results combined, we made a radar graph for each mouse strain (figure 5). In the radar graphs the complete control group (0/0) (blue field) is plotted against the complete TDI-treated group (1/1) (red field). The radar graphs visualize pronounced differences between Th2- and Th1-biased mouse strains. While BALB/c, BP2 and A/J mice gave high values in respiratory, inflammatory and immunological parameters characteristic for occupational asthma, C57Bl/6 and CBA mice showed hardly any responses in our mouse model. In the context of ‘a mouse model of chemical-induced asthma’ both the limited response of the 0/0 group in the BALB/c mice, along with a pronounced response in the 1/1 group, makes the BALB/c mouse strain the most favorable to use, at least in the short term. When we approach the results from a different angle, we can also suggest that C57Bl/6 mice could be a good mouse strain to investigate why most people do not develop allergy and asthma, while some do. The radar graphs show also that TDI sensitization yields a mixed Th1-Th2 profile, as previously shown by us and other research groups, especially in the Th2-biased mouse strains [10], [12], [25], [31], [32]. Therefore, in the context of chemical-induced asthma, it is not correct to state that a certain mouse strain is more Th2-biased than Th1-biased.

In conclusion, this study, based on a comparison of seven different inbred mouse strains in a model of chemical-induced asthma, demonstrates that the genetic background of the different mouse strains has a large impact on the phenotypical outcome of TDI-induced asthma. Caution has to be taken when comparing results from different mouse strains. Furthermore, in this model, BALB/c mice represented best the characteristics of chemical-induced asthma.
